# Deep Learning-Based Mapping of Tumor Infiltrating Lymphocytes in Whole Slide Images of 23 Types of Cancer

**DOI:** 10.3389/fonc.2021.806603

**Published:** 2022-02-16

**Authors:** Shahira Abousamra, Rajarsi Gupta, Le Hou, Rebecca Batiste, Tianhao Zhao, Anand Shankar, Arvind Rao, Chao Chen, Dimitris Samaras, Tahsin Kurc, Joel Saltz

**Affiliations:** ^1^ Department of Computer Science, Stony Brook University, Stony Brook, NY, United States; ^2^ Department of Biomedical Informatics, Stony Brook University, Stony Brook, NY, United States; ^3^ Department of Pathology, Stony Brook University, Stony Brook, NY, United States; ^4^ Department of Computational Medicine & Bioinformatics, University of Michigan, Ann Arbor, MI, United States

**Keywords:** TIL maps, digital histopathology, whole slide images, tumor infiltrating lymphocytes, deep learning, large scale analysis

## Abstract

The role of tumor infiltrating lymphocytes (TILs) as a biomarker to predict disease progression and clinical outcomes has generated tremendous interest in translational cancer research. We present an updated and enhanced deep learning workflow to classify 50x50 um tiled image patches (100x100 pixels at 20x magnification) as TIL positive or negative based on the presence of 2 or more TILs in gigapixel whole slide images (WSIs) from the Cancer Genome Atlas (TCGA). This workflow generates TIL maps to study the abundance and spatial distribution of TILs in 23 different types of cancer. We trained three state-of-the-art, popular convolutional neural network (CNN) architectures (namely VGG16, Inception-V4, and ResNet-34) with a large volume of training data, which combined manual annotations from pathologists (strong annotations) and computer-generated labels from our previously reported first-generation TIL model for 13 cancer types (model-generated annotations). Specifically, this training dataset contains TIL positive and negative patches from cancers in additional organ sites and curated data to help improve algorithmic performance by decreasing known false positives and false negatives. Our new TIL workflow also incorporates automated thresholding to convert model predictions into binary classifications to generate TIL maps. The new TIL models all achieve better performance with improvements of up to 13% in accuracy and 15% in F-score. We report these new TIL models and a curated dataset of TIL maps, referred to as *TIL-Maps-23*, for *7983* WSIs spanning *23* types of cancer with complex and diverse visual appearances, which will be publicly available along with the code to evaluate performance.

**Code Available at:** https://github.com/ShahiraAbousamra/til_classification.

## 1 Introduction

Tumor infiltrating lymphocytes (TILs) have gained importance as a biomarker in translational cancer research for predicting clinical outcomes and guiding treatment. As our collective understanding of tumor immune responses in cancer expands, clinical research studies have shown that high densities of TILs correlate with favorable clinical outcomes (1), such as longer disease-free survival (2) and/or improved overall survival in multiple types of cancer (3). Studies also suggest that the spatial distribution of TILs within complex tumor microenvironments may play an important role in cancer prognosis (4–6). These findings have led to efforts to characterize the abundance and spatial distribution of TILs in cancer tissue samples to further our understanding of tumor immune interactions and help develop precision medicine applications in oncology (7–11).

Computational image analysis of whole slide images (WSIs) of cancer tissue samples has become a very active area of translational biomedical research. The goals are to gain novel insights into cancer and the tumor microenvironment, including tumor immune responses, through the search for biomarkers to predict outcomes and treatment response. Modern digital microscopes scan whole slide tissue samples at very high image resolutions, ranging from 50,000x50,000 pixels to over 100,000x100,000 pixels. The increasing availability of such gigapixel WSIs has stimulated the development of image analysis methods for detection, segmentation, and classification of microanatomic regions, structures, cells, and other objects in tissue images. Therefore, we utilized advances in computer vision and machine learning to quantitatively characterize TILs to complement qualitative microscopic evaluation of cancer tissue samples by pathologists. Deep learning has become the preferred approach for a variety of image analysis tasks in recent years (12–17) since these methods can analyze raw image data and do not require specified instructions to identify and quantify engineered image features. Furthermore, deep learning-based image analysis workflows have been shown to consistently produce more accurate results and generalize to new datasets better than previous image analysis methods in computational pathology.

Several projects have implemented methods to detect and classify lymphocytes in tissue images. Eriksen et al. (18) employed a commercial system to count CD3+ and CD8+ cells in tissue images that were obtained from stage II colon cancer patients and stained with an immunohistochemistry (IHC) protocol. Swiderska-Chadaj (19) also trained a deep learning model with a dataset of 171,166 annotated CD3+ and CD8+ cells in images of IHC stained tissue specimens from breast, prostate and colon cancer cases. Garcia et al. (20) proposed a deep learning model to count TILs in IHC images of gastric cancer tissue samples by using a model trained with 70x70 square pixel patches extracted from biopsy micrographs scanned at 40x magnification and labeled by pathologists. PathoNet, developed by Negahbani et al. (21), implements a deep learning model based on the U-Net architecture (22) for detection and classification of Ki-67 and TILs in breast cancer cases.

Methods were also developed to study TILs in Hematoxylin and Eosin (H&E) stained tissue images. Budginaite et al. (23) developed a deep learning workflow based on the Micro-Net architecture (24) and multi-layer perceptrons to identify lymphocytes in tissue images from breast and colorectal cancer cases. Corredor et al. (25) investigated the spatial patterns of TILs in early stage non-small cell lung cancer cases with the goal of predicting cancer recurrence. Jaber et al. (26) investigated TILs in non-small cell lung cancer cases by employing deep learning architectures and support vector machines to classify 100x100 square micron patches in WSIs. Acs et al. (27) developed a computerized TIL scoring method using QuPath software (28) to cluster melanoma cancer patients into those with favorable prognosis and those with poor prognosis. Linder et al. (29) evaluated the use of deep learning for TIL analysis in tissue images of testicular germ cell tumors by using commercial image analysis software and implementing a two stage workflow in which the first stage processed WSIs to detect regions that contained TILs and the second stage counted the TILs in those regions, demonstrating how deep learning-based methods can be used successfully for TIL detection in germ cell cancer. Amgad et al. (30) proposed a deep learning workflow based on a fully convolutional network architecture developed by Long et al. (31) to identify tumor, fibroblast, and lymphocyte nuclei and tumor and stroma regions. Le et al. (32) developed deep learning models for segmentation of tumor regions and detection of TIL distributions in whole slide images of breast cancer tissues by training models based on VGG16, Inception-V4, and Resnet-34 architectures that used WSIs from The Surveillance, Epidemiology, and End Results (SEER) Program at the National Cancer Institute (NCI) and the Cancer Genome Atlas (TCGA) repository.

**Figure 1:**
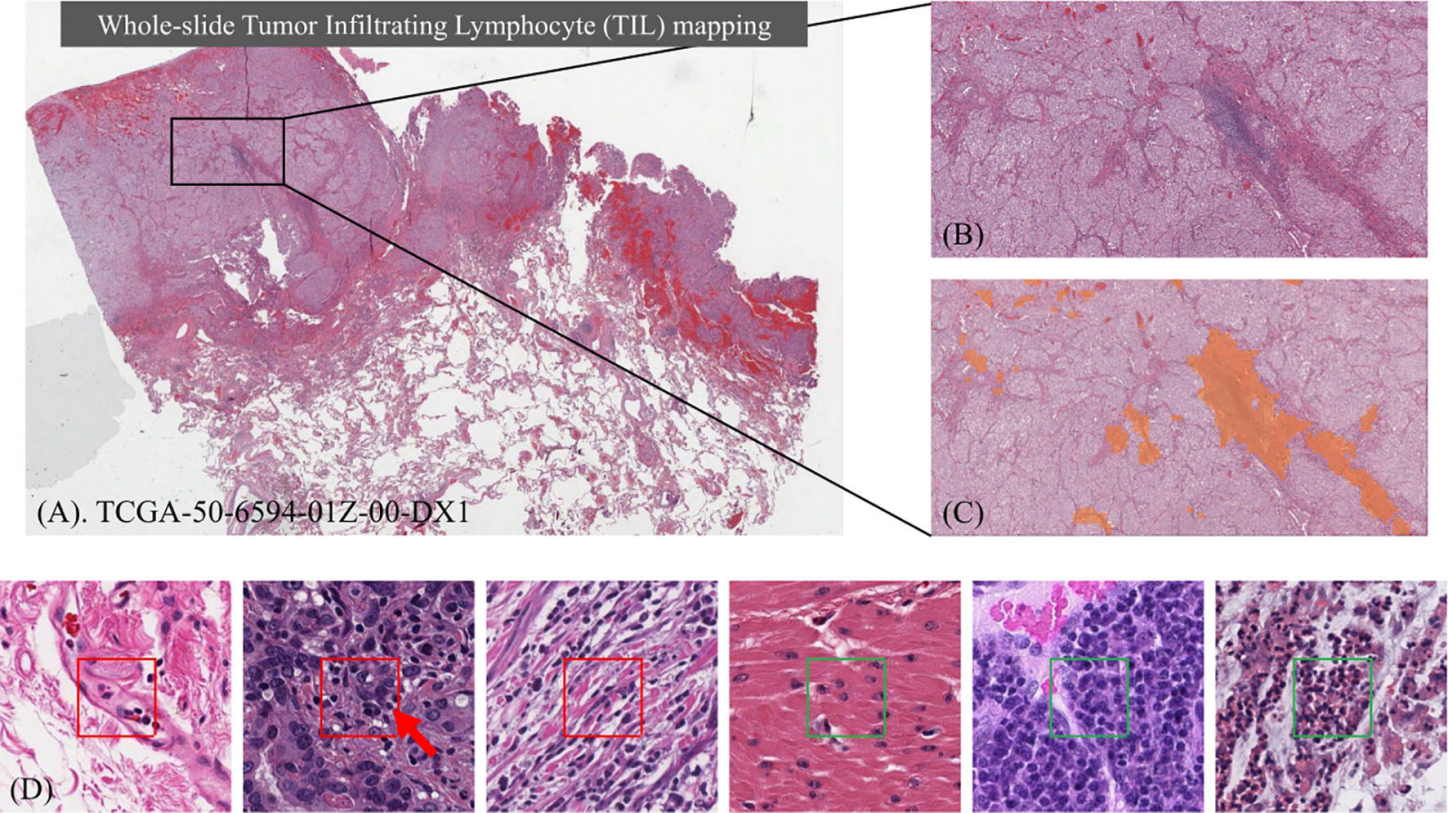
Identifying Tumor Infiltrating Lymphocyte (TIL) regions in gigapixel pathology WSIs. **(A)** H&E stained WSI of lung adenocarcinoma. **(B)** Example of a region of tissue. **(C)** Example of a TIL map overlaid on the region of tissue. **(D)** Examples of TIL positive (framed in red) and negative (framed in green) patches. A lymphocyte is typically dark, round to ovoid, and relatively small compared to tumor and normal nuclei. Sample patches show the heterogeneity in TIL regions and how it can be challenging to differentiate TIL positive and TIL negative regions.

Despite an increasing number of projects, there are few large scale datasets of WSIs that are publicly available to study TILs. Moreover, most of the previous projects targeted specific types of cancer from particular organ sites. The classification of TILs can be challenging in large datasets of WSIs across multiple types of cancer from different organ sites for many reasons. Deep learning models need to distinguish TILs from cancer cells that are intrinsically complex across a wide spectrum of growth patterns, cellular and nuclear morphologies, and other histopathologic features associated with specific types of cancer, which vary by organ site, state of cellular differentiation, and stage of cancer (e.g. primary organ site versus a metastatic tumor deposit). Computational image analysis of pathology WSIs is also complicated by variations in image properties from differences in scanning with different types of digital slide scanners and varying tissue staining laboratory protocols. **Figure 1** shows an example of identifying TILs in a WSI and the heterogeneity of the appearance and distribution of TILs in different tissue samples. Before our work, the largest TIL dataset was generated by Saltz et al. (33), where 5202 WSIs from 13 cancer types were analyzed.

In this paper we describe a deep learning workflow that was utilized to generate a large dataset of TIL maps, referred to here as the TIL-Maps-23 dataset. Unlike the previous work that studied TILs in mostly common types of cancer, we trained a deep learning model with the goal of analyzing WSIs from a much wider range of different types of cancer. We adopted the same approach of patch-wise classification as in (33), where each WSI is partitioned into non-overlapping patches of size 50 x 50 square microns. A trained deep learning CNN model classifies each patch as TIL-positive or TIL-negative and then compiled to generate a TIL map of the WSI. While a classification at the cellular level allows finer grain analysis, patch-level classification offers several advantages. First, it requires much less annotation time and effort. The pathologist can just mark regions as TIL positive or TIL negative and then we can sample patches from these regions. On the other hand, cell-level annotations require marking each individual lymphocyte cell in a patch. Second, optimizing nuclear classification is more challenging over multiple cancer types and needs much more data. Our approach allows us to scale the dataset to develop a model to span more cancer types with much less effort. Third, the identifying lymphocytes at a 50 microns resolution provides valuable and interpretable information about the spatial distributions of TILs across large sets of WSIs to study many samples from a particular type of cancer and/or compare the role of TILs in different types of cancer, which can be further studied in downstream correlative analyses. In an earlier work (33), we applied spatial statistics to patch-level TIL predictions in WSIs and demonstrated that spatial clustering patterns of TILs correlate with molecular features and clinical outcomes. In another work (32), we computed TIL infiltration amounts by combining patch-level TIL predictions with tumor segmentation results in breast cancer and showed correlations between TIL infiltration and survival that was stratified by molecular subtype.

The work presented in this manuscript focuses on an improved deep learning workflow for patch-level TIL prediction and generation of a large dataset of TIL predictions across multiple cancer types. We plan to carry out additional studies to ascertain the clinical relevance of TIL predictions in future works. Our work improves on the earlier work done by Saltz et al. (33) in several ways. The previous work trained two CNN deep learning models, one for detecting lymphocytes and the other for segmenting necrosis regions by using convolutional neural networks (CNNs) developed in-house. The necrosis segmentation model was used to eliminate false TIL-positive predictions in necrotic regions of tissues, which required two separate training datasets. This new and improved deep learning workflow employs a single CNN by adapting popular, engineered classification networks and using a combination of manual annotations and machine-generated annotations as training data. Moreover, the previous work included a manual thresholding step in order to generate the final binary TIL maps. This step consisted of a patch sampling process and a manual review of the sampled patches to set TIL-positive/TIL-negative thresholds for different WSIs. The new workflow implements an automated mechanism for computing thresholds to map model predictions to binary classifications. This eliminates the manual thresholding step of the previous work. After all of these improvements, we present the TIL-Maps-23 dataset for 23 types of cancer, which is the largest collection of curated TIL maps across both common and rare types of cancer to date.

## 2 Materials and Methods

**Figure 2:**
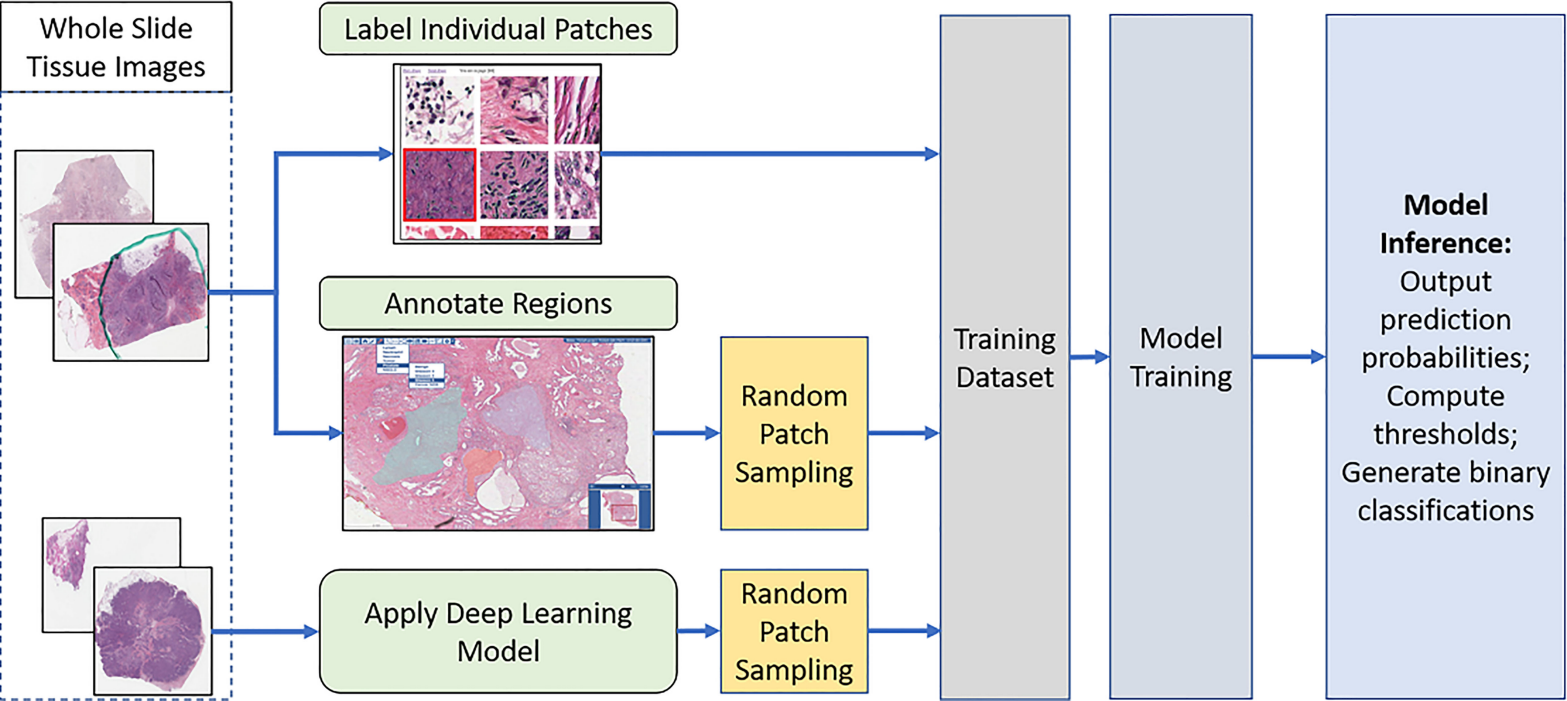
The overall analysis workflow: Training data generation, model training, inference (prediction), and computing threshold values. Training data is generated *via* a combination of manual annotations and model-generated predictions. A trained model generates predictions in the form of the probability that a patch is TIL-positive. The probability values are mapped to binary classifications by applying an automatically computed threshold.

**Figure 3:**
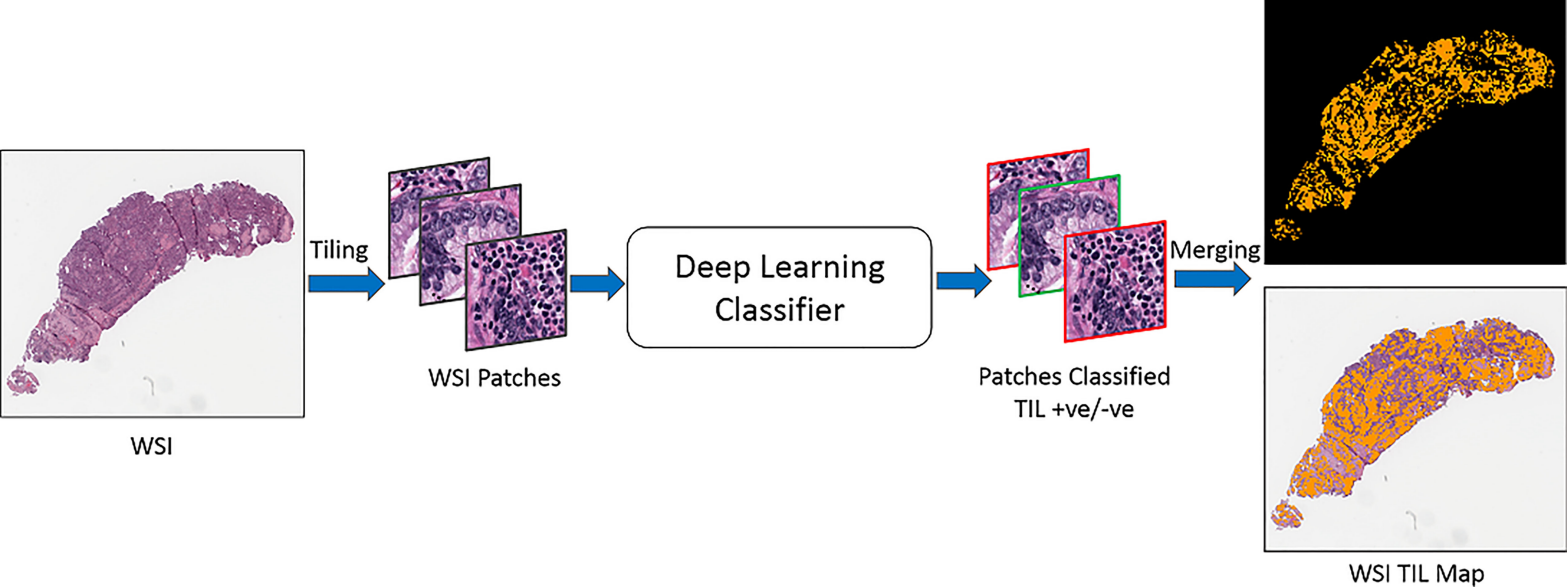
TIL inference step. An input WSI is partitioned into disjoint patches of 50x50 square microns. Each patch is processed by the trained classification model and assigned a probability value. The probability values are mapped to binary classifications. A TIL map covering the entire WSI is generated.

The overall analysis workflow is illustrated in **Figure 2**. The workflow consists of training data generation, model training, and inference steps. The training dataset is generated by combining labels from manual patch-level and region-level annotations, as well as classification predictions generated by the deep learning model developed in (33). The inference step (**Figure 3**) partitions WSIs into patches, outputs patch-level probability values, and executes an automated method to compute thresholds for mapping the probability values to binary classifications.

### 2.1 Generating Training Dataset

We created a training dataset by combining manually annotated patches (strong annotations) from 18 TCGA cancer types (ACC, BRCA, COAD, ESCA, HNSC, KIRC, LIHC, LUAD, MESO, OV, PAAD, PRAD, SARC, SKCM, TGCT, THYM, UCEC, and UVM) and model-generated annotations from 4 TCGA cancer types (CESC, LUSC, READ, and STAD). For the model-generated annotations, we sampled a set of patches classified by the model in (33). The model-generated annotations are employed not only as a cost-saving mechanism to reduce manual annotation workload but also to increase diversity in texture and appearance of tissue data. Variations in texture and appearance are often the case with H&E images, especially with a dataset like TCGA which comes from multiple sites, each using their own slide scanners and staining protocols. We have shown previously in (34) that combining manual annotations with model-generated annotations for cancer types with scarce or no manual annotations gives better results compared to using manual annotations alone.

The manual annotations are generated in 2 ways. First, patches of 150 x 150 square microns are randomly sampled from the WSIs. Pathologists annotate the center 50 x 50 square micron sub-patch in each patch. The annotation indicates whether the center sub-patch is TIL-positive or TIL-negative. Using a 150 x 150 square micron patch allows pathologists to see the surrounding tissue for a more informed decision on the label of the center sub-patch. Only the center sub-patch is used in training. A patch is labeled TIL-positive if it has at least 2 lymphocytes or plasma cells in the center sub-patch. Second, pathologists mark TIL-positive and TIL-negative regions on WSIs, where TIL-positive regions are regions with a significant amount of lymphocytes and/or plasma cells. Patches of 50 x 50 square microns are randomly sampled from these regions, where each patch is assigned the same label as the source region.

The model-generated annotations are collected from classifications produced by the previous model in (33). This model employed a human-in-the-loop TIL classification procedure, where a manual threshold step was applied to the predicted TIL probability maps in order to produce binary classifications. In our work, we randomly sampled TIL-positive and TIL-negative patches from the binary classifications.

### 2.2 Deep Neural Network Models and Training

We trained 3 models with different networks: VGG-16 (35), ResNet-34 (36), and Inception-V4 (37). These networks are engineered for image classification. They have been shown to be powerful classifiers on the ImageNet dataset (38) and have been adopted in various computer vision applications. The main differences between the 3 networks can be summarized as follows: VGG-16 has a basic convolutional neural network architecture; ResNet-34 is much deeper and features skip connections that allow a more stable training of the deeper network; and Inception-v4 is an even a deeper network, where each block in the network utilizes residual connections and convolutional layers of various sizes to capture features at different resolutions and reception fields.

Each network is initialized with weights from the respective pre-trained model on ImageNet. The batch normalization layers are dropped. Each input image (patch) is scaled with bilinear interpolation to match the network’s pre-training input size (i.e., 224 x 224 pixels for VGG-16, 299 x 299 pixels for Inception-V4, and 100 x 100 for ResNet-34). The input image is normalized to the range [–1, 1] for VGG-16 and Inception-V4 by $img=\left(\frac{img}{255}-0.5\right)\times 2$ . For ResNet-34, the input image is normalized with the same mean and standard deviation vectors as the pre-trained model. The training phase implements data augmentation, including random rotation and flipping, shifting of input patches left/right and up/down by a random number of pixels in the range of [–20, +20], and color augmentation *via* small variations to brightness and color in the hue, saturation, and lightness (HSL) space. All of the networks were trained end-to-end using the cross entropy loss.

### 2.3 Determining Binary Classification Thresholds

The trained models output a probability value for each patch in an input WSI. This creates a probability map for the entire WSI. The final binary prediction (TIL positive or TIL negative) is obtained by thresholding the probability map. If the probability of a patch is greater than or equal to the threshold value, the patch is classified as TIL-positive. Otherwise, it is classified as TIL-negative.

**Figure 4:**
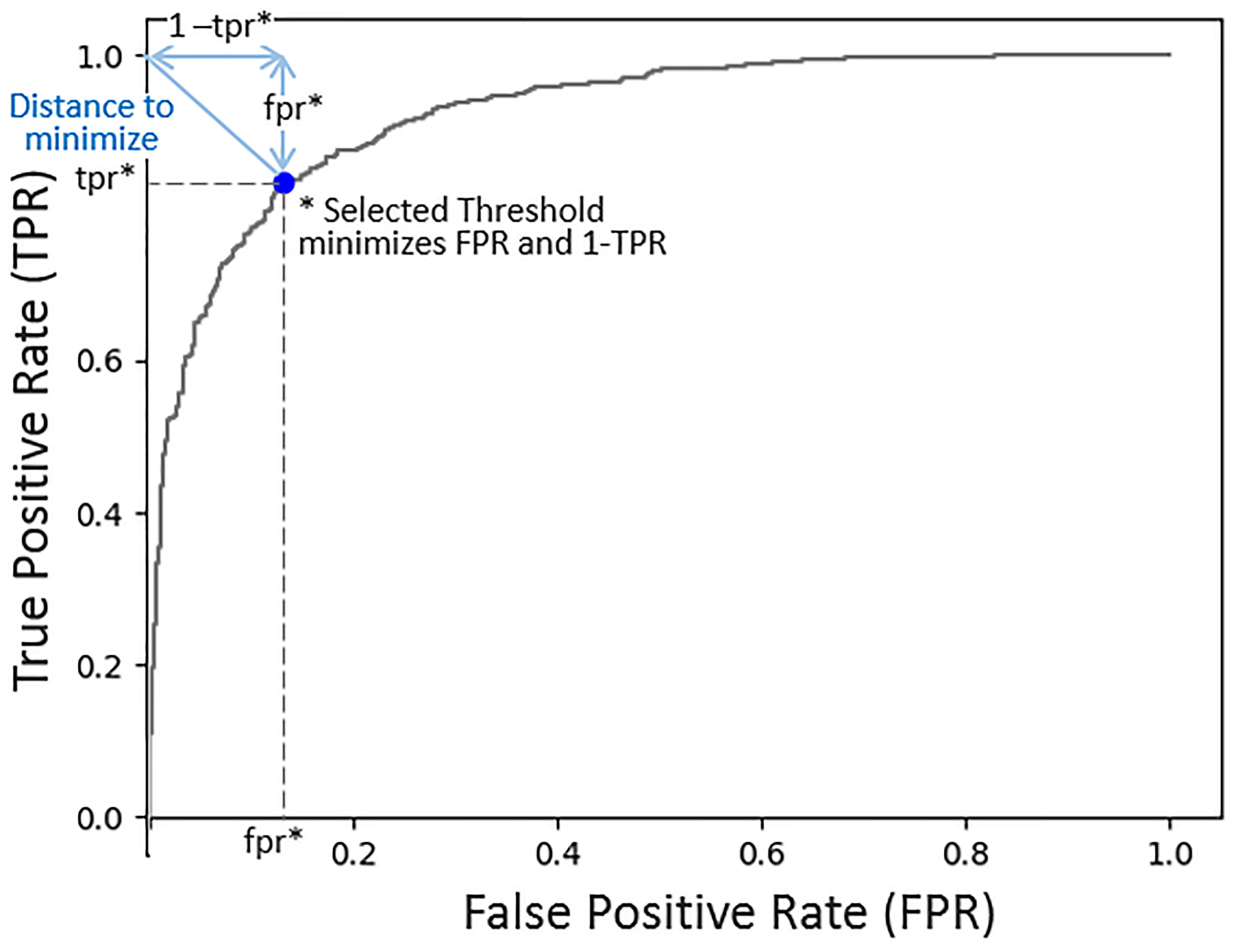
Model probability threshold selection. The objective is to minimize the false positive rate (FPR) and maximize the true positive rate (TPR). This ROC plot (FPR, TPR), illustrates that by minimizing the distance from the point (0,1) to the curve, we are minimizing FPR^2^ + (1 **-** TPR)^2^, thus achieving our objective. The selected threshold corresponds to the FPR and TPR at which the line intersects with the curve minimizing the distance to the point (0,1).

A default threshold value of 0.5 was used during training to evaluate a model’s performance in each training epoch. At the end of the training phase, the threshold value was fine-tuned for the inference phase. A threshold value in the range [0.4, 0.6] was selected for each model based on the performance of the model on a small *hold-out* dataset. We evaluated two methods for selecting the threshold value for each model. The first method relies on the true positive rate (TPR) and the false positive rate (FPR) (39). The optimal (FPR, TPR) pair is (0,1). The threshold selection method minimizes the FPR and maximizes the TPR. **Figure 4** shows an example receiver operating characteristic (ROC) curve (*x* = *FPR*, *y* = *TPR*). The length of the line from the (0,1) point and intersecting the curve at (fpr, tpr) is $\sqrt{{\text{fpr}}^{2}+{\left(1-\text{tpr}\right)}^{2}}$ . By selecting the threshold value that minimizes the distance from (0,1) to the curve, FPR and (1-TPR) are minimized. The second method is based on the Youdin Index, which is commonly used to select a threshold that maximizes TPR - FPR (40). In our experiments, both methods resulted in almost identical binary classification maps. The threshold values selected for the VGG-16, ResNet-34, and Inception-V4 models were 0.4, 0.56, and 0.41, respectively.

### 2.4 Software Support for Training Data Generation and Review of Analysis Results

The WSIs in the image dataset are loaded to a software platform, called Quantitative Imaging in Pathology (QuIP), for training data generation and review of the model predictions. QuIP consists of multiple services, implemented as micro-services with software containers, and a set of Web-based applications that support viewing of WSIs, annotation of image regions and patches, and interactive viewing of model predictions as heatmaps overlaid on WSIs (41).

One of the web applications is a markup and annotation tool with multiple class label selections (**Figure S2** in supplementary material). This tool enables annotations of full-resolution whole slide tissue images. The user can draw a polygon to mark up a region and select a label from a pull-down menu to label the region. Multiple regions and classes can be annotated in an image. In addition to marking regions, pathologists can annotate individual patches. Another web application is used for this purpose. A set of patches are displayed to the user who can assign a label to each patch by clicking on the patch. To minimize the number of mouse clicks (or taps on touch screens) for the binary classification case, we assume a default class for all patches. The user clicks on patches that belong to the alternative class only.

**Figure 5:**
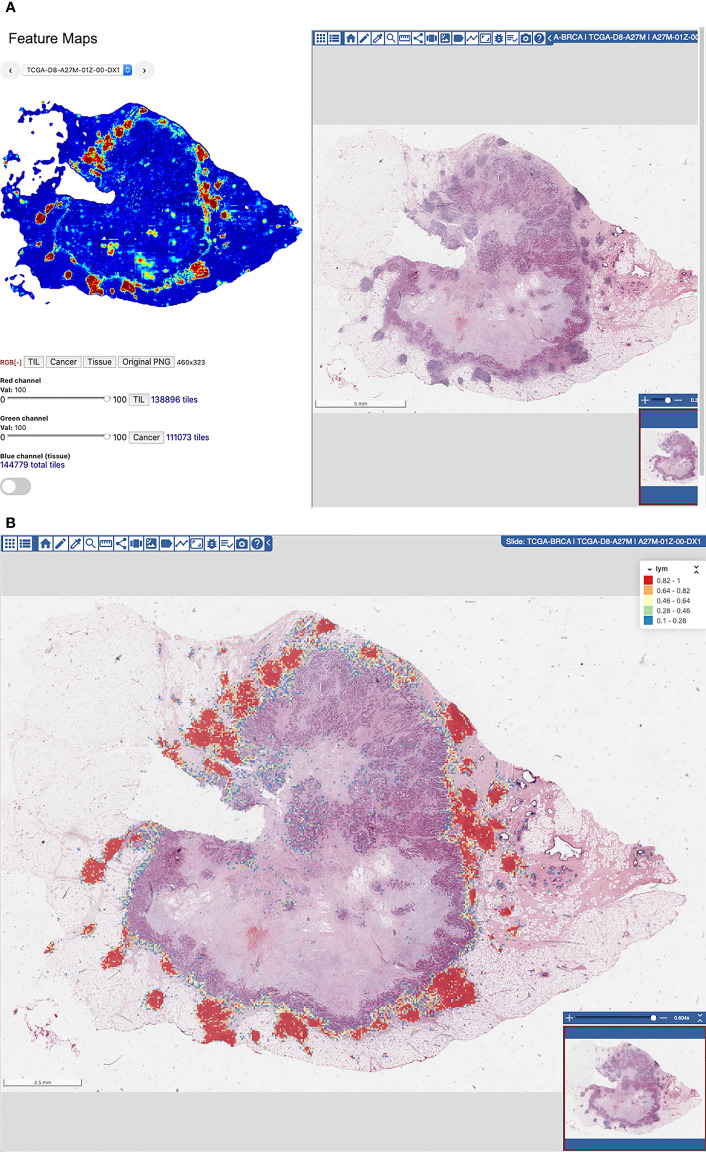
**(A)** FeatureMap along with a view of the tissue image. **(B)** Heatmap viewer and editor for viewing of heatmaps on full-resolution WSIs and for fine-grain re-labeling of patches to generate additional training data.

Manual examination of model predictions requires interactive interrogation and visual analytic tools that link these results with the underlying images. QuIP implements two tools for this purpose; the FeatureMap tool and the heatmap viewer/editor. The FeatureMap tool converts probability maps into low resolution heatmaps, called featuremaps, which can be visualized at a lower image resolution than at the resolution of whole slide images (**Figure 5A**). Each pixel in a featuremap image corresponds to a patch in the WSI. The goal is to let a user rapidly go through a set of images without having to load heatmaps on full-resolution images and pan and zoom in the images. After reviewing a featuremap, the user can click anywhere on the featuremap image and visualize the region at full image resolution using the heatmap viewer/editor. The heatmap viewer/editor allows a user to access full-resolution heatmap representation of a probability map overlaid on the input WSI and re-label algorithm predictions (**Figure 5B**). The user can click on an area in a heatmap, zoom and pan, and interactively examine the areas of interest. If the user determines that predictions in some areas should be corrected, the user switches to the heatmap editor and annotates a set of patches to be positive or negative on the WSI. The FeatureMap and heatmap viewer/editor tools rely on the backend data management and indexing services of QuIP, namely PathDB for managing images and FeatureMap data and FeatureDB for managing probability maps and user annotations.

### 2.5 Evaluating Model Performance

We evaluated the performances of the trained models *via* two methods: patch-level classification accuracy and region categorical classification performance.

For patch-level classification accuracy, we collected manually labeled test patches and measured the performance of each model with these patches using the accuracy and F-score metrics. The accuracy metric represents the percent of correctly classified patches and is computed as:


(1) $$Accuracy=\frac{TP+TN}{TP+TN+FP+FN}\times 100\%$$

Here TP is the number of true positives, TN is the number of true negatives, FP is the number of false positives, and FN is the number of false negatives. The F-score measures the balance of model precision and how many of the positive patches are correctly classified (i.e. recalled). It is computed as:


(2) $$Precision=\frac{TP}{TP+FP},\;Recall=\frac{TP}{TP+FN},\;F-score=\frac{2\times Precision\times Recall}{Precision+Recall}$$

For the region categorical classification performance, we adopted the evaluation method implemented in (33). We evaluated the correlation between predictions from the models and annotations (labels) from the pathologists, both quantitatively and qualitatively using *super-patches*. Super-patches make it easier to collect a large number of annotations from multiple pathologists. This evaluation method provides a higher level of evaluation that is beyond individual patches and offers a quantification of the correlation between a model’s predictions and a pathologist’s perception of TIL distribution.

**Figure 6:**
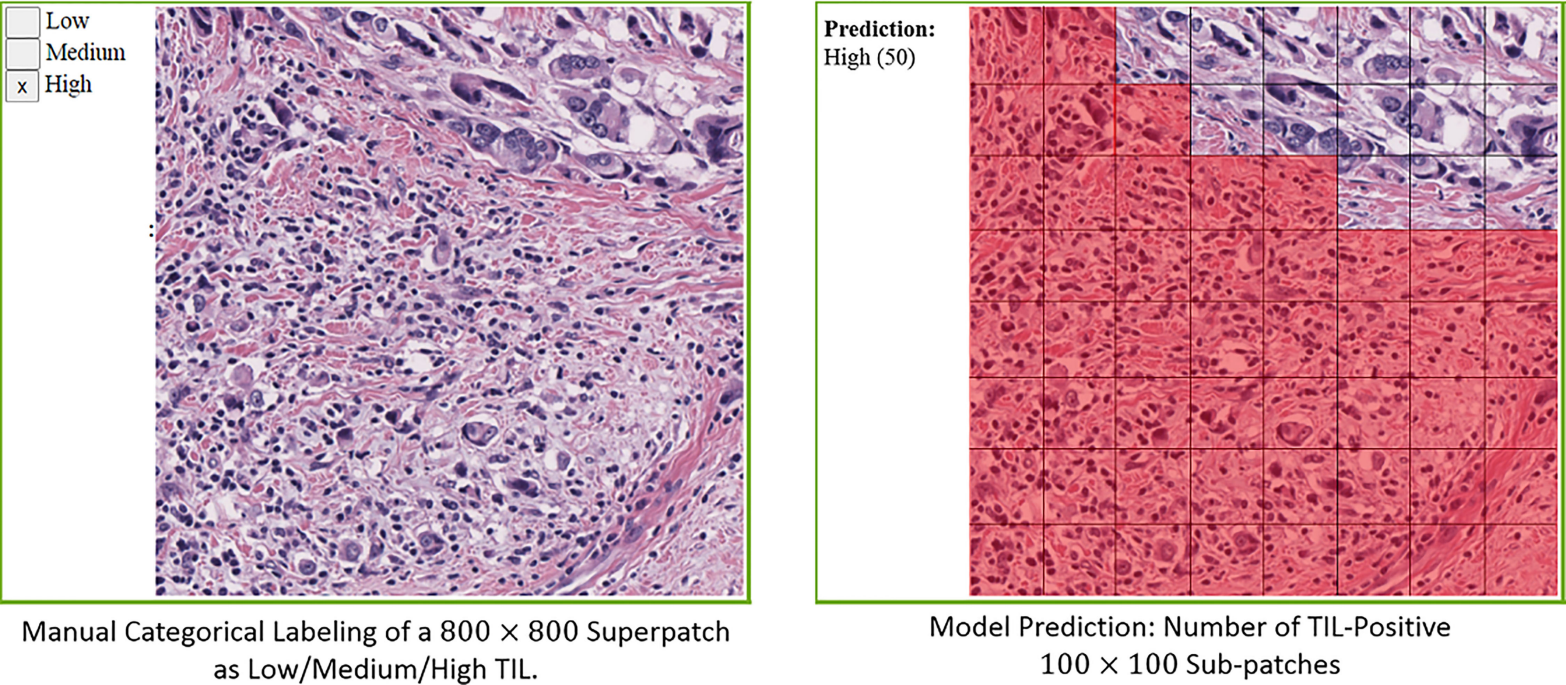
Illustration of a superpatch labeling and prediction.

A super-patch is defined as a large 800 x 800 square pixel patch at 20x magnification (i.e., a super-patch covers a 400 x 400 square micron area in tissue). The deep learning models classify 100 x 100 square pixel patches at 20x magnification. Hence, each super-patch is divided into an 8 x 8 grid, and each patch (of 100 x 100 square pixels) is classified as TIL-Positive or TIL-Negative. **Figure 6** shows an example of a super-patch and the labeling of its patches.

In our work, each super-patch was annotated by one to three pathologists as Low TIL, Medium TIL, or High TIL, based on the perceived fraction of the area of the TIL-positive patches. The *score* of a deep learning model for a given super-patch is the number of patches classified as TIL-positive by the model. Hence, each super-patch gets assigned a score between 0 to 64.

We use the polyserial correlation method (42, 43) to quantify the correlation between the model scores and the pathologist annotations. Polyserial correlation measures the inferred latent correlation between a continuous variable and an ordered categorical variable, which, in our case, represent scoring by the model and the rounded average TIL-positive annotations from the pathologists, respectively. We also used violin plots for the qualitative evaluation of the correlation between the model scoring and the pathologists’ categorical labels. Violin plots can be viewed as box plots that show the smoothed probability density distribution rotated on each side.

## 3 Results

### 3.1 Dataset and Implementation Details

The number of patches in training and test sets are given in **Tables S1** and **S2** in the supplementary material. On average, 19 WSIs per cancer type were used in manually annotated training data and 117 WSIs per cancer type were used in model-generated training annotations. There were 351,272 patches in total in the training dataset. Out of these patches, 282,065 were manually annotated and 69,207 were patches from the model-generated annotations dataset. The model-generated annotations allowed us to reduce the manual annotation effort by 19% and increase training data diversity by covering 22 cancer types (the training dataset did not include patches from BLCA), while maintaining a good ratio of strong annotations to model-generated annotations.

We trained three models with popular networks, namely Inception V4 (37), VGG-16 (35) and ResNet-34 (36), as described in Section 2. The models were trained with the Adam optimizer using a learning rate of 0.00005 and a batch size of 128.

### 3.2 Patch-Level Classification Accuracy

We collected 327, 299, 326, and 299 of manually labeled test patches from BRCA, LUAD, SARC, and OV, respectively, and 888 patches in total from the other cancer types with 47 patches per cancer type on average. **Tables 1** and **2** show the accuracy and F-score, respectively, for the three models, as well as the model trained in (33), referred to as the *Baseline* model in the tables. The columns LUAD, BRCA, SARC, and OV show the performance numbers in each metric for the patches collected from these four cancer types. The columns *Other* and *All* show the performance values with the 888 patches from the other cancer types and with all of the patches, respectively. The column *13 Cancer Types* shows the performance comparison between the Baseline model and the newer models with patches from the 13 cancer types (BLCA, BRCA, CESC, COAD, LUAD, LUSC, PAAD, PRAD, READ, SKCM, STAD, UCEC, and UVM) analyzed in the previous work (33). The results show that the new models outperformed the Baseline model by up to 13% in accuracy and 15% in F-score. All of the new models performed well, attaining high accuracy and F-score values. In most of the cases, the Inception V4 model achieved better performance, in the range of 1–5% higher values, than the other models.

**Table 1:** Evaluation of patch classification accuracy.

Model Name	LUAD	BRCA	SARC	OV	Other*	13 cancer types**	All
Baseline	73.60%	74.90%	–	–	–	79.56%	–
VGG-16	83.28%	**88.38%**	94.17%	88.29%	82.52%	83.32%	86.02%
ResNet-34	84.28%	86.24%	91.41%	87.29%	82.10%	82.45%	85.14%
Incep-V4	**86.29%**	87.16%	**96.93%**	**94.31%**	**82.53%**	**83.68%**	**87.43%**

Compare result for each of LUAD, BRCA, SARC, OV, *Other: patches from other cancer types in the set of 23 types used in training, **13 cancer types: subset of test patches belonging to the 13 cancer types the baseline model with human in the loop (Baseline) (33) was trained on, All: all test patches from all the 23 cancer types. Best accuracy in each dataset is indicated in bold.

**Table 2:** Patch classification F-score results.

Model Name	LUAD	BRCA	SARC	OV	Other*	13 cancer types**	All
Baseline	0.78	0.77	–	–	–	0.85	–
VGG-16	0.85	0.88	0.92	0.84	0.85	0.86	0.86
ResNet-34	0.87	0.87	0.88	0.82	0.86	0.86	0.86
Incep-V4	**0.89**	**0.89**	**0.96**	**0.93**	**0.87**	**0.88**	**0.89**

Compare result for each of LUAD, BRCA, SARC, OV, *Other: patches from other cancer types in the set of 23 types used in training, **13 cancer types: subset of test patches belonging to the 13 cancer types the baseline model with human in the loop (Baseline) (33) was trained on, All: all test patches from all the 23 cancer types. Best F-score in each dataset is indicated in bold.

### 3.3 Region Categorical Classification

**Figure 7:**
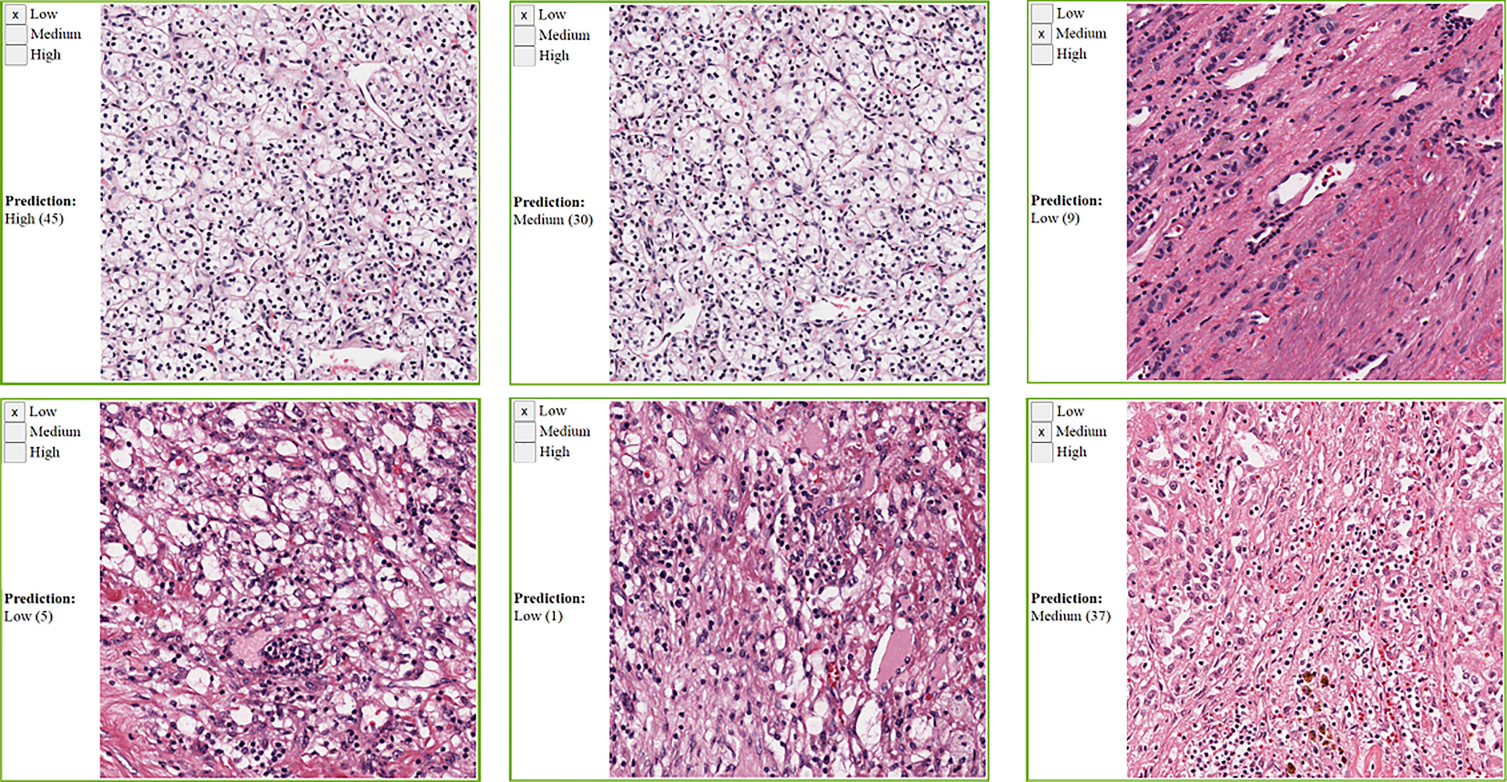
Sample KIRC super-patches, showing the categorical label and the Inception model prediction. KIRC is challenging because other cell types nuclei can look like lymphocytes. The model prediction is displayed as a category and a score between brackets. The models’ scoring is a value in the range 0 to 64. We roughly interpret it as: Low if 0 ≤score≤ 21, Medium if 22 ≤score ≤ 42, and High otherwise. Top row: cases where the category approximated from the model scoring does not match the pathologists’ label. Bottom row: cases where the category approximated from the model scoring matches the pathologists’ label.

**Figure 8:**
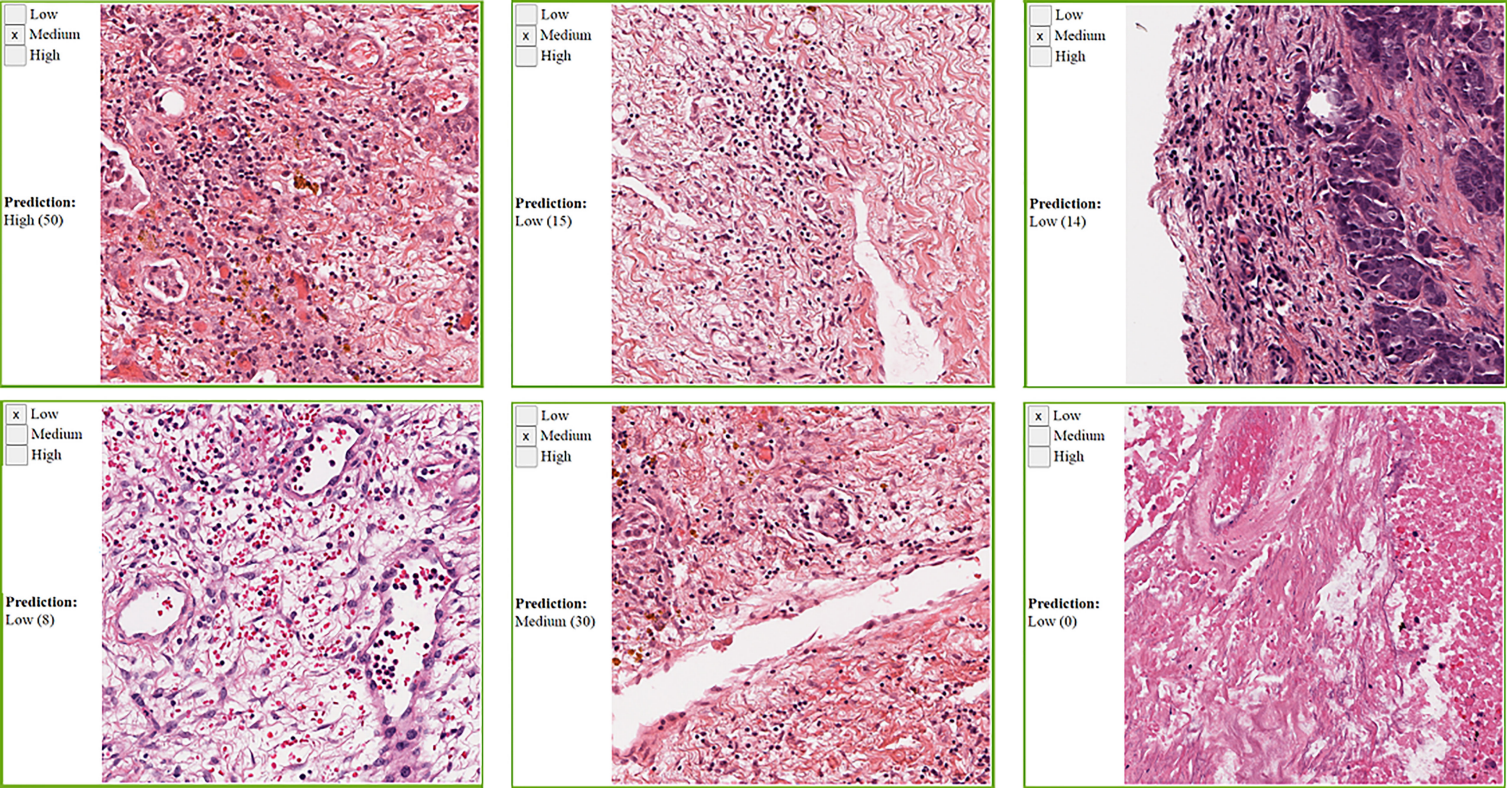
Sample OV super-patches, showing the categorical label and the Inception model prediction. The model prediction is displayed as a category and a score between brackets. The models’ scoring is a value in the range 0 to 64. We roughly interpret it as: Low if 0 ≤core ≤ 21, Medium if 22 ≤core ≤ 42, and High otherwise. Top row: cases where the category approximated from the model scoring does not match the pathologists’ label. Bottom row: cases where the category approximated from the model scoring matches the pathologists’ label.

We collected manual annotations on 4,198 randomly selected super-patches from the 23 cancer types. **Table 3** shows the polyserial correlation coefficient for each model for super-patches from individual cancer types. The last column in the bottom set of the table is the polyserial correlation coefficient with respect to the collective set of super-patches and the mean and standard deviation over the correlation coefficients of the individual cancer types. The results show that no single model is consistently better than the other models. The Inception V4 model achieves a higher mean score as shown in the *ALL* column of the table. The correlation coefficients are the lowest for KIRC. The nuclei of cells in KIRC are generally small, dark, and rounded, which gives the tumor cells a similar appearance to lymphocytes. Thus, the deep learning models classify them incorrectly and overestimate TIL regions. **Figure 7** shows some of the super-patches that were incorrectly scored by the Inception V4 model. The left panel in the figure shows the categorical label (Low, Medium and High) of the super-patch assigned by the pathologists as well as the model prediction and the number of patches classified as TIL-positive by the model in parentheses. For the sake of presentation in the figure, the model prediction is described as Low, if the model score is 0 ≤score ≤ 21, Medium if the score is 22 ≤score ≤ 42, and High >42. Similar low correlations were obtained with super-patches from OV. The Inception V4 model resulted in under-estimation in 14 cases versus over-estimation in 9 cases of the OV super-patches. **Figure 8** shows various sample results from the model with the OV super-patches, illustrating the discrepancy between the model scoring and the pathologists’ classifications. The polyserial correlation coefficient is greater than or equal to 0.8 for 13 cancer types (ACC, BRCA, ESCA, HNSC, LIHC, MESO, PAAD, PRAD, READ, SARC, SKCM, TGCT, and UVM), between 0.7 and 0.8 for 5 cancer types (LUSC, THYM, STAD, BLCA, and UCEC) and below 0.7 for 5 cancer types (COAD, CESC, OV, LUAD, and KIRC).

**Table 3:** Superpatches evaluation using polyserial correlation coefficient.

Model Name	ACC (147)	BLCA (64)	BRCA (348)	CESC (61)	COAD (65)	ESCA (312)	HNSC (324)	KIRC (319)
Baseline	–	0.720	0.552	**0.679**	0.329	–	–	–
VGG-16	0.879	**0.787**	0.745	0.592	0.688	0.777	**0.904**	0.515
ResNet-34	0.925	0.740	0.797	0.654	0.658	**0.810**	0.883	**0.599**
Incep-V4	**0.963**	0.744	**0.797**	0.667	**0.695**	0.805	0.897	0.598
**Model Name**	**LIHC (248)**	**LUAD (63)**	**LUSC (65)**	**MESO (271)**	**OV (158)**	**PAAD (440)**	**PRAD (66)**	**READ (62)**
Baseline	–	0.615	0.658	–	–	0.695	0.819	0.706
VGG-16	**0.891**	0.670	**0.830**	**0.840**	0.565	**0.886**	**0.885**	0.702
ResNet-34	0.872	**0.733**	0.775	0.805	0.527	0.874	0.862	0.715
Incep-V4	0.854	0.617	0.789	0.818	**0.635**	0.870	0.818	**0.811**
**Model Name**	**SARC (299)**	**SKCM (67)**	**STAD (63)**	**TGCT (303)**	**THYM (324)**	**UCEC (64)**	**UVM (64)**	**ALL (4198)**
Baseline	–	0.666	0.728	–	–	0.692	0.681	–
VGG-16	0.912	0.816	0.713	**0.859**	0.774	0.667	0.896	0.807 (0.77 ± 0.12)
ResNet-34	**0.932**	0.794	**0.821**	0.799	0.765	**0.766**	0.899	0.808 (0.78 ± 0.10)
Incep-34	0.921	**0.822**	0.752	0.823	**0.790**	0.742	**0.913**	**0.820 (0.79 ± 0.10)**

The number in brackets indicated the number of superpatches in the respective cancer type. Baseline is the model developed in (33).

Highest polyserial correlation in each dataset (cancer type) is indicated in bold.

**Figure 9:**
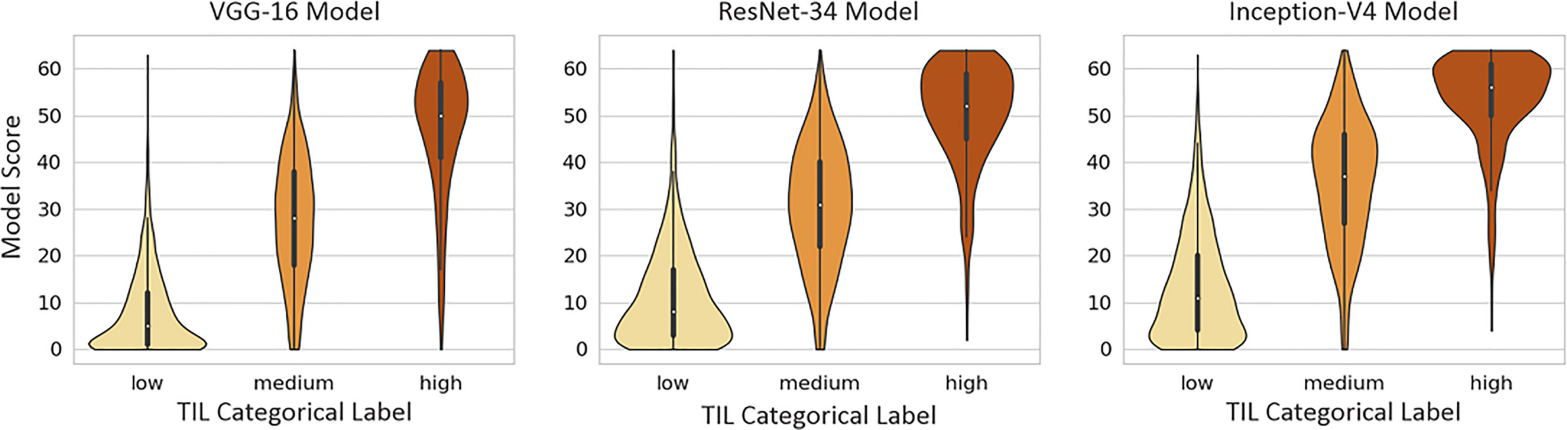
Violin plots of each model’s scores against super-patch categorical labels (Low, Medium, and High TIL).

**Figure 9** shows the violin plots for scores from each deep learning model against the rounded average of pathologists’ annotations. The visual representations of the density distributions and the median values indicate that the VGG-16 model tends to under-estimate TILs. The ResNet-34 and Inception-V4 models are more consistent with the pathologist categorical labeling, where the Inception-V4 model performs better.

### 3.4 TIL Area Estimation

After we evaluated the performance of these TIL models and visually confirmed how well TILs were being classified in WSIs across 23 types of cancer, the next step was to utilize the best TIL model to analyze all of the available diagnostic DX1 TCGA WSIs in these types of cancer to characterize the abundance and spatial distribution of TILs as a potential biomarker. Based on our evaluations, we utilized the Inception model to analyze all diagnostic DX1 TCGA WSIs since it had the highest patch classification accuracy and F-score and best overall performance on the super-patches. We used the Inception-V4 TIL model to generate all of the TIL maps in this dataset and compute the estimated average area that is infiltrated by TILs per WSI in the dataset across 23 types of cancer. The results are summarized in **Table 4** and demonstrate how computational pathology is very useful in characterizing TILs as a biomarker, which can be very helpful in guiding future clinical research in precision oncology and immunotherapy by supporting cohort discovery by identifying potential types of cancer with high abundance of intra- and peri-tumoral TILs.

**Table 4:** Estimated percent TIL area (mean±standard deviation) across WSIs in the dataset TIL-Maps-23.

Cancer Type	TIL Area	Cancer Type	TIL Area	Cancer Type	TIL Area
ACC	1.96 ± 5.15	BLCA	8.60 ± 8.23	BRCA	6.37 ± 7.38
CESC	15.69 ± 11.57	COAD	9.60 ± 6.62	ESCA	11.34 ± 8.45
HNSC	13.54 ± 10.36	KIRC	6.74 ± 8.43	LIHC	7.80 ± 8.27
LUAD	14.29 ± 11.31	LUSC	15.59 ± 10.29	MESO	7.64 ± 8.03
OV	3.94 ± 4.96	PAAD	10.42 ± 7.78	PRAD	5.73 ± 6.52
READ	9.04 ± 6.23	SARC	6.44 ± 9.28	SKCM	13.42 ± 14.46
STAD	15.29 ± 13.24	TGCT	14.51 ± 14.19	THYM	52.89 ± 26.88
UCEC	7.87 ± 8.40	UVM	2.20 ± 2.34	–	–

## 4 Discussion

We described and evaluated a deep learning workflow that creates TIL maps to facilitate the quantitative characterization of TILs and map their spatial distributions in H&E WSIs of cancer tissue specimens. Since H&E staining is routinely performed for diagnostic histopathologic evaluation of tissue samples, we developed this workflow to analyze TILs in H&E WSIs that are becoming more commonly available as digital pathology is being more commonly adopted in clinical laboratories. Studies have shown that the host immune system is capable of controlling tumor growth through the activation of adaptive and innate immune surveillance mechanisms (44) and that the spatial context and nature of cellular heterogeneity of the tumor microenvironment are important in cancer prognosis (1, 4, 45, 46). This has led to TILs becoming important in the clinical arena with increasing importance in precision medicine (47–49). Thus, having the ability to quantify TILs in diagnostic H&E WSIs of tissue images is becoming incredibly important as we collectively expand our understanding about tumor immune interactions and their role in disease progression, recurrence, treatment response, and survival.

Therefore, our goal was to develop a robust computational pathology workflow for H&E WSIs to reliably characterize TILs in the tumor microenvironment in a uniform manner. We generated TIL maps to complement traditional microscopic examination so that pathologists and research scientists could interpret the abundance and distribution of TILs alongside the assessment of invasive growth patterns and other histopathologic features across 23 types of cancer. The interest in harnessing the power of TILs to fight cancer continues to grow with advances in immunotherapy, chemoradiation regimens, and other treatment modalities, which has led to important translational cancer research initiatives by the International Immuno-Oncology Biomarker Working Group in creating standardized visual reporting guidelines for pathologists to evaluate TILs in breast cancer and other solid tumors (49–54). Even though pathologists can follow the guidelines and perform qualitative and semi-quantitative assessments of TILs in cancer, the task is highly challenging, subjective, and prone to intra- and interobserver variability. Our results show that the new TIL models are quite useful for both qualitative and quantitative evaluation of TILs in WSIs. The TIL maps are also very useful for discerning how much of the tissue samples contain mononuclear lymphoplasmacytic infiltrates and their spatial distribution in individual cancer tissue samples and across several different kinds of cancer from various organ sites. And most importantly, these new models perform better than the model developed in the earlier work, which was limited to 13 different types of cancer (33).

We attribute the better results to the use of state-of-the-art engineered networks and our larger and more diverse training dataset that includes both computer-generated annotations and manual annotations. Having the capability to computationally analyze WSIs to study fascinating patterns of tumor immune interactions with reliable and reproducible methods represents a highly significant opportunity for cancer research to help improve cancer treatment and clinical management. This novel data about the quantity and distribution TILs from H&E WSIs is also important as a biomarker for downstream correlative prognostic studies with clinical, radiologic, laboratory, molecular, and pharmacologic data. Moreover, these kinds of analyses facilitate large-scale research to elucidate deeper mechanistic understanding of the role of tumoral immunity in disease progression and treatment response across both common and rarer types of cancer. Furthermore, the identification and quantification of other image features would allow for the formulation of higher-order relationships to explore the role of TIL infiltrates in cancer immunology with respect to histologic patterns of tumor growth, tumor grade, tumor heterogeneity, cancer recurrence, and metastasis.

In this work, we used three popular network architectures, VGG16, Inception V4, and ResNet-34, to train models for the detection and classification of TILs in tissue images. There are other state-of-the-art networks, such as Xception (55) and EfficientNet (56), which have shown excellent performance in image classification tasks. Our choice of the networks is primarily based on the fact that we have used these selected networks for other projects. Since deep learning is a rapidly evolving field, future work will explore incorporating other deep learning architectures into our workflow to further improve performance and expand the capabilities and applicability of our workflow. We utilized our models to generate TIL maps, referred to here as the *TIL-Maps-23* dataset, in 7983 H&E WSIs in 23 tumor types in the TCGA data repository from among approximately 12,000 diagnostic WSIs from 33 cancer types.

The *TIL-Maps-23* dataset covers 70% of the TCGA cancer types and 67% of the diagnostic TCGA WSIs. Beyond the information embedded in pathology WSIs, the TCGA dataset also includes demographic, clinical, and molecular data derived from multiple molecular platforms, which presents a readily available opportunity to integrate image-derived features, such as TIL-tumor distance distributions or TIL spatial cluster distributions, with rich molecular and clinical data to gain a more comprehensive understanding about tumor immune interactions and the role of TILs as a biomarker. To the best of our knowledge, this is the largest set of TIL maps to date. The list of cancer types included in the dataset is in **Table 5**. In addition to making our models and Tensorflow CNN codes publicly available, we are also releasing the dataset of TIL maps with the intention of motivating translational cancer research and algorithmic development for image analysis in computational pathology.

**Table 5:** The list of cancer types in TIL-Maps-23, the number of WSIs for each cancer type, and the polyserial correlation coefficients for the Inception-V4 model, sorted in descending order.

Cancer Type	# WSIs	Polyserial Correlation Coefficient
Adrenocortical carcinoma (ACC)	323	0.96
Sarcoma (SARC)	255	0.92
Uveal melanoma (UVM)	80	0.91
Head and Neck squamous cell carcinoma (HNSC)	450	0.90
Pancreatic adenocarcinoma (PAAD)	189	0.87
Liver hepatocellular carcinoma (LIHC)	365	0.85
Mesothelioma (MESO)	175	0.82
Prostate adenocarcinoma (PRAD)	403	0.82
Skin cutaneous melanoma (SKCM)	448	0.82
Testicular germ cell tumors (TGCT)	154	0.82
Esophageal carcinoma (ESCA)	156	0.81
Rectum adenocarcinoma (READ)	165	0.81
Breast invasive carcinoma (BRCA)	1068	0.80
Lung squamous cell carcinoma (LUSC)	484	0.79
Thymoma (THYM)	121	0.79
Stomach adenocarcinoma (STAD)	434	0.75
Bladder urothelial carcinoma (BLCA)	386	0.74
Uterine corpus endometrial carcinoma (UCEC)	506	0.74
Colon adenocarcinoma (COAD)	453	0.69
Cervical squamous cell carcinoma (CESC)	268	0.67
Ovarian serous cystadenocarcinoma (OV)	106	0.64
Lung adenocarcinoma (LUAD)	480	0.62
Kidney renal clear cell carcinoma (KIRC)	514	0.60

## 5 Conclusion

The growth of cancer immunotherapy has created tremendous interest in characterizing the abundance and spatial distribution of TILs in cancer tissue samples in order to explore their clinical significance to help guide treatment. As the footprint of Digital Pathology rapidly expands in translational cancer research and clinical laboratories with the recent FDA approval of whole slide imaging for primary diagnostic use, it is widely expected that a large majority of pathology slides will be routinely digitized within the next 5-10 years. In parallel, advances in machine learning, computer vision, and computational hardware resources have led to an increased focus on deep learning-based techniques for segmentation and classification of various features of tissue microanatomy in WSIs, including regions, microanatomic structures, cells, nuclei, and other features. The characterization of TIL infiltrated tissue in WSIs at a resolution of 50 microns by using our methods goes far beyond what can be reproducibly and scalably observed by human beings across hundreds and thousands of tissue samples. Tools and methodologies that augment or enable such characterizations can improve the practice of pathology while we march towards realizing the goal of precision oncology.

## Data Availability Statement

The datasets presented in this study can be found in online repositories. The names of the repository/repositories can be found below: https://stonybrookmedicine.box.com/v/til-results-new-model.

## Author Contributions

SA, RG, LH, CC, DS, TK, and JS contributed to the design of the deep learning workflow. SA implemented the workflow and carried out the experiments for evaluation. SA, RG, LH, AS, AR, and JS designed the experimental evaluation. RG, RB, and TZ contributed to the data annotation. SA, RG, JS, and TK led the generation of the TIL-Maps-23 dataset. TK, JS, and RG led the development of the software for training data generation and management and visualization of images and TIL maps. SA, RG, CC, DS, TK, and JS edited the manuscript. All authors contributed to the article and approved the submitted version.

## Funding

This work was supported by the National Institutes of Health (NIH) and National Cancer Institute (NCI) grants UH3-CA22502103, U24-CA21510904, 1U24CA180924-01A1, 3U24CA215109-02, and 1UG3CA225021-01 as well as generous private support from Bob Beals and Betsy Barton. AR and AS were partially supported by NCI grant R37-CA214955 (to AR), the University of Michigan (U-M) institutional research funds and also supported by ACS grant RSG-16-005-01 (to AR). AS was supported by the Biomedical Informatics & Data Science Training Grant (T32GM141746). This work was enabled by computational resources supported by National Science Foundation grant number ACI-1548562, providing access to the Bridges system, which is supported by NSF award number ACI-1445606, at the Pittsburgh Supercomputing Center, and also a DOE INCITE award joint with the MENNDL team at the Oak Ridge National Laboratory, providing access to Summit high performance computing system. The funders were not involved in the study design, collection, analysis, interpretation of data, the writing of this article or the decision to submit it for publication.

## Conflict of Interest

The authors declare that the research was conducted in the absence of any commercial or financial relationships that could be construed as a potential conflict of interest.

## Publisher’s Note

All claims expressed in this article are solely those of the authors and do not necessarily represent those of their affiliated organizations, or those of the publisher, the editors and the reviewers. Any product that may be evaluated in this article, or claim that may be made by its manufacturer, is not guaranteed or endorsed by the publisher.
