# Supplementary material for: Deep Learning-Based Mapping of Tumor Infiltrating Lymphocytes in Whole Slide Images of 23 Types of Cancer
Source: Front Oncol. 2022 Feb 16;11:806603. doi: 10.3389/fonc.2021.806603 (PMC8889499; doi:10.3389/fonc.2021.806603)
Supplement: Supplementary file 1 [file DataSheet_1.pdf]

Deep Learning-based Mapping of Tumor Infiltrating Lymphocytes in Whole Slide Images of 23 Types of Cancer - Supplementary Material

1 SUPPLEMENTARY FIGURES

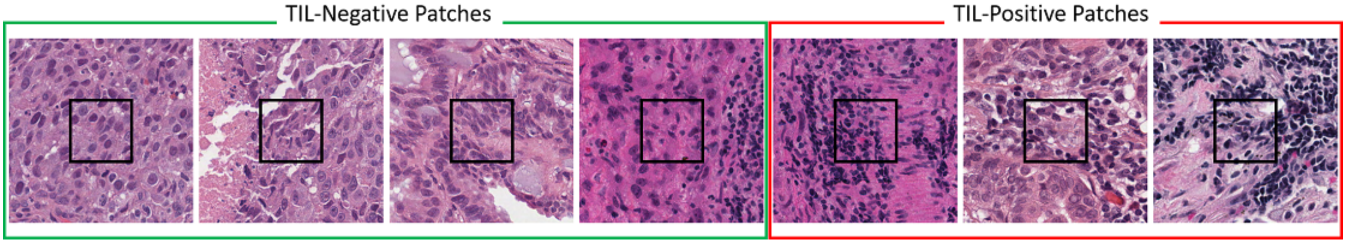

Figure S1. Samples of manually annotated patches. They are labeled TIL-positive if there are lymphocytes or plasma cells present in the center square region (sub-patch). Only the sub-patch is used in training. The larger area provides context to pathologists during annotation.

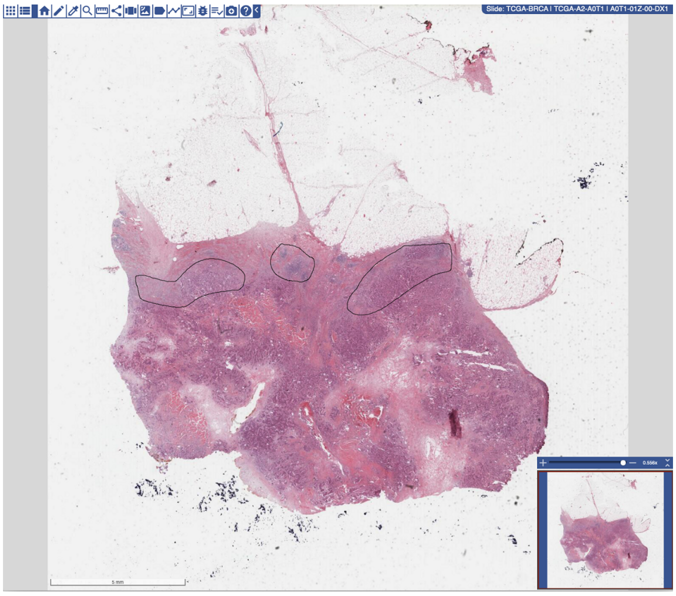

Figure S2. Markup and annotation tool that can be used to annotate multiple regions and classes in a whole slide tissue image. These annotations can be used to create training datasets for deep learning classification models.

2 SUPPLEMENTARY TABLES

Table S1 show the number of training patches per cancer type and Table S2 shows the number of patches and superpatches per cancer type in the test set.

Cancer Type	TIL Pos.	TIL Neg.	Total	Cancer Type	TIL Pos.	TIL Neg.	Total
ACC	573	31952	32525	BRCA	1674	4930	6604
CESC	3116	10028	13144	COAD	2035	1947	3982
ESCA	4245	10637	14882	HNSC	4748	12136	16884
KIRC	2017	27300	29317	LIHC	2014	14645	16659
LUAD	5233	27142	32375	LUSC	6406	19218	25624
MESO	1283	9517	10800	OV	651	21184	21835
PAAD	1121	4452	5573	PRAD	686	4842	5528
READ	1210	3533	4743	SARC	1310	18538	19848
SKCM	10548	23515	34063	STAD	6743	16515	23258
TGCT	2347	16205	18552	THYM	4235	2983	7218
UCEC	1267	4153	5420	UVM	39	2399	2438

Table S1. The number of labelled positive and negative patches per cancer type in the training set.

Cancer Type	Patches Count	Superpatches Count	Cancer Type	Patches Count	Superpatches Count
ACC	20	147	BRCA	327	348
CESC	57	61	COAD	78	65
ESCA	-	312	HNSC	13	324
KIRC	63	319	LIHC	61	248
LUAD	299	63	LUSC	76	65
MESO	-	271	OV	299	158
PAAD	80	440	PRAD	67	66
READ	69	62	SARC	326	299
SKCM	82	67	STAD	72	63
TGCT	-	303	THYM	-	324
UCEC	65	64	UVM	-	66

Table S2. The number of labelled patches and superpatches in the test set.
